# 2-Oxo-Imidazole-Containing Dipeptides Play a Key Role in the Antioxidant Capacity of Imidazole-Containing Dipeptides

**DOI:** 10.3390/antiox10091434

**Published:** 2021-09-08

**Authors:** Shingo Kasamatsu, Somei Komae, Kana Matsukura, Yuki Kakihana, Koji Uchida, Hideshi Ihara

**Affiliations:** 1 Department of Biological Science, Graduate School of Science, Osaka Prefecture University, 1-1 Gakuen-cho, Sakai 599-8531, Osaka, Japan; kasamatsu@b.s.osakafu-u.ac.jp (S.K.); sbc04036@edu.osakafu-u.ac.jp (S.K.); sbc04099@edu.osakafu-u.ac.jp (K.M.); swc04032@gmail.com (Y.K.); 2 Graduate School of Agricultural and Life Sciences, The University of Tokyo, Tokyo 113-8657, Japan; a-uchida@mail.ecc.u-tokyo.ac.jp

**Keywords:** 2-oxo-imidazole-containing dipeptides, imidazole-containing dipeptides, carnosine, anserine, 2-oxo-carnosine, 2-oxo-anserine, antioxidant capacity, DPPH assay, FRAP assay, ORAC assay

## Abstract

There is substantial evidence for the antioxidant functions of imidazole-containing dipeptides (IDPs), including carnosine and anserine, under physiological and pathological conditions in vivo. However, the detailed mechanism underlying the antioxidant functions is still poorly understood. Recently, we discovered the endogenous production of 2-oxo-imidazole-containing dipeptides (2-oxo-IDPs), such as 2-oxo-carnosine and 2-oxo-anserine, as novel derivatives of IDPs in mouse tissues and revealed that the antioxidant capacity of 2-oxo-carnosine was much greater than that of carnosine. However, the antioxidant capacity of 2-oxo-IDPs still remains unclear. In this study, we evaluated 2-oxo-carnosine and 2-oxo-anserine by multiple in vitro assays, such as 2,2-diphenyl-1-picrylhydrazyl (DPPH) radical scavenging, ferric reducing/antioxidant power, and oxygen radical absorbance capacity assays in comparison with the corresponding IDPs, carnosine and anserine. All the assays employed herein demonstrated that 2-oxo-carnosine and 2-oxo-anserine exhibited a greater antioxidant capacity than that of the corresponding IDPs. Quantitative high-performance liquid chromatography tandem mass spectrometry revealed that commercial IDPs standards were contaminated with a certain amount of 2-oxo-IDPs, which was correlated with the antioxidant capacity. DPPH radical scavenging assay revealed that the elimination of contaminated 2-oxo-IDPs from the IDPs standards caused a significant decrease in the antioxidant capacity compared to the original IDPs standards. These results suggest that the main driver of the antioxidant capacity of IDPs is 2-oxo-IDPs; accordingly, the conversion of IDPs to 2-oxo-IDPs may be a critical step in the antioxidant functions.

## 1. Introduction

**Figure 1:**
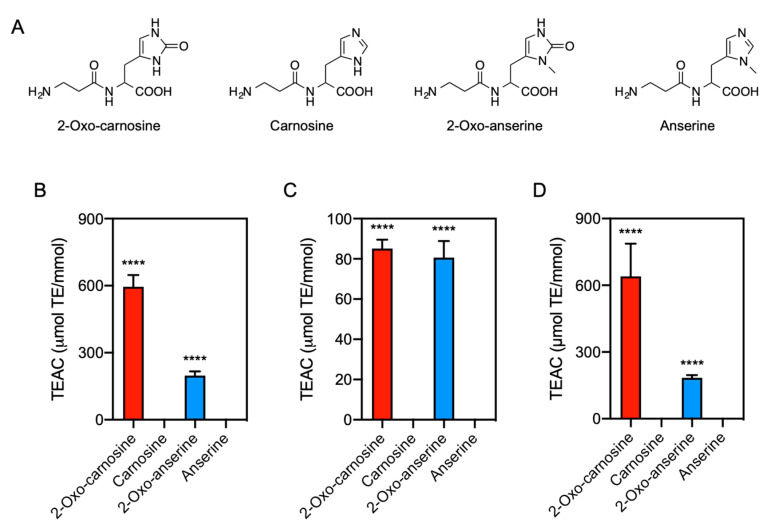
Antioxidant capacity of 2-oxo-IDPs and IDPs. (**A**) Molecular structures of 2-oxo-carnosine, carnosine, 2-oxo-anserine, and anserine. (**B**–**D**) Antioxidant capacity of 2-oxo-IDPs and IDPs was evaluated by DPPH radical scavenging assay (**B**), ferric reducing/antioxidant power assay (**C**), and oxygen radical absorbance capacity assay (**D**). Data are expressed as Trolox equivalent antioxidant capacity (TEAC): µmol Trolox equivalent (TE) per mmol samples. Data are presented as means ± standard error of the mean (SEM) (*n* > 4). **** *p* < 0.0001 versus the corresponding IDPs, compared using unpaired Student’s *t* test.

Imidazole-containing dipeptides (IDPs) is the collective term for dipeptides containing an imidazole residue. Since the discovery of carnosine (β-alanyl-l-histidine) in beef extract in 1900 [1], the endogenous production of several IDPs, such as anserine (β-alanyl-3-methyl-l-histidine) and homocarnosine (γ-aminobutyryl-l-histidine), has been reported (Figure 1A) [2]. Various vertebrates, including mammals, produce IDPs, with wide variation in the composition and concentration [3]. IDPs is particularly abundant in the skeletal muscle and brain; however, IDPs has also been detected in other tissues, such as the heart and kidney [4]. IDPs is endogenously generated by several enzymes, including carnosine synthase and carnosine *N*-methyltransferase [5,6]. In addition, IDPs is degraded by carnosinase but not by common dipeptidases [7]. Therefore, IDPs is relatively stable in vivo. Thus, the homeostasis of IDPs is tightly regulated by specific enzymes in vivo, suggesting that they have important physiological functions.

Numerous studies have reported that IDPs exhibits several biological functions (reviewed in [2]). In particular, their antioxidant capacity has been a major focus of research since it was first reported in the 1980s [8]. IDPs can scavenge reactive oxygen species (ROS), such as superoxide anions [9] and hydroxyl radical (HO^•^) [10], nitric oxide (NO) [11], and reactive nitrogen species such as peroxynitrite (ONOO^−^) [12], and hypochlorous acid (HClO) [13,14]. In addition, cell and animal experiments have shown that IDPs exerts protective effects against various oxidative stress-related diseases, such as cancer, neurodegenerative disease, diabetes, and aging [3]. However, the antioxidant capacities of IDPs evaluated by in vitro assays are considerably lower than those of other well-known endogenous antioxidants, such as glutathione and ascorbic acid [15]. The reasons for the discrepancies in the antioxidant capacities of IDPs remain unclear.

We have recently discovered that 2-oxo-imidazole-containing dipeptides (2-oxo-IDPs), including 2-oxo-carnosine, 2-oxo-anserine, 2-oxo-homocarnosine, and 2-oxo-homoanserine, is endogenously produced as a novel oxidized derivative of IDPs (Figure 1A) [4,16]. 2-oxo-IDPs is produced under normal conditions in several mouse tissues, and an increase in their production was observed in the brain tissue of oxidative stress model mice [4]. Notably, a 2,2-diphenyl-1-picrylhydrazyl (DPPH) radical scavenging assay revealed that 2-oxo-carnosine exhibited a much greater antioxidant capacity than did its precursor, carnosine [4]. Moreover, 2-oxo-carnosine significantly protected SH-SY5Y neuroblastoma cells from rotenone-induced oxidative stress, whereas no cytoprotective effects of carnosine were observed [4]. These findings strongly suggest that 2-oxo-carnosine contributes to the biological functions, including the antioxidant function, of carnosine. However, the functions of other 2-oxo-IDPs, such as 2-oxo-anserine, remain unclear, and further detailed analyses of the biological functions of IDPs and 2-oxo-IDPs are still needed.

In this study, we aimed to evaluate the overall antioxidant capacity of 2-oxo-IDPs (2-oxo-carnosine and 2-oxo-anserine) and IDPs (carnosine and anserine) using multiple in vitro antioxidant assays, including DPPH radical scavenging, ferric reducing/antioxidant power (FRAP), and oxygen radical absorbance capacity (ORAC) assays. For the careful evaluation of the genuine antioxidant capacity of IDPs, contaminated 2-oxo-IDPs was removed from commercial IDPs standards using high-performance liquid chromatography (HPLC) and a DPPH assay with highly purified IDPs was performed.

## 2. Materials and Methods

### 2.1. Materials

l-Carnosine was obtained from the following sources: Biosynth AG (Staad, Switzerland; 99% purity), Wako Pure Chemical Industries (Osaka, Japan; 98% purity), Peptide Institute, Inc. (Osaka, Japan; 99% purity) and Sigma-Aldrich (St. Louis, MO, USA; 99% purity). l-Anserine was obtained from Toronto Research Chemicals (North York, ON, Canada; 98% purity) and Ark Pharm, Inc. (Arlington Heights, IL, USA; 97% purity). l-Anserine nitrate was obtained from Angene (London, UK; 98% purity) and BLD Pharmatech Ltd. (Shanghai, China; 95% purity). 2-oxo-carnosine and 2-oxo-anserine were prepared as described previously [4].

Trolox and DPPH were obtained from Merck (Darmstadt, Germany), Tokyo Chemical Industry (Tokyo, Japan), respectively. 2,2’-Azobis(2-methylpropionamidine) dihydrochloride (AAPH) and fluorescein were obtained from Wako Pure Chemical Industries. All other chemicals and reagents were obtained from common suppliers and were of the highest grade commercially available. 

### 2.2. Measurement of Antioxidant Capacity

The DPPH radical scavenging assay was carried out as previously described [4,17], with slight modifications. The DPPH stock solution was prepared in ethanol. In brief, in a 96-well plate, 250 μM DPPH was incubated in 30 mM sodium phosphate buffer (pH 7.4) in the presence or absence of 2-oxo-IDPs or IDPs (final 2.5–40 μM) at room temperature (25 °C) for 20 min unless otherwise noted. Absorbance at 517 nm was measured using an Infinite 200 PRO microplate reader (Tecan, Männedorf, Switzerland). Trolox solutions (final 2.5–40 μM) were used for defining the standard curve. Radical scavenging capacity was evaluated by the inhibition ratio (%), which was calculated by the following formula: Inhibition ratio (%) = (Ac − As)/Ac × 100, where Ac and As indicate the absorbances of the blank control (water) and sample, respectively [4,17]. The radical scavenging capacity was expressed as Trolox equivalent antioxidant capacity (TEAC), which was calculated using the Trolox standard curve.

The FRAP assay was carried out as described previously [18], with a slight modification. In brief, FRAP solution was freshly prepared by mixing 300 mM sodium acetate buffer (pH 3.6), 10 mM 2,4,6-tris(2-pyridyl)-1,3,5-triazine (TPTZ, Dojindo Laboratories, Kumamoto, Japan), and 20 mM FeCl_3_ at a volume ratio of 10:1:1. In a 96-well plate, FRAP solution (180 μL) was incubated in the dark at 37 °C for 30 min prior to the addition of 5 μL of 2-oxo-IDPs or IDPs (final 1.6–50 μM). After 15 min incubation at 37 °C, absorbance at 596 nm was measured by an Infinite 200 PRO microplate reader. Trolox (final 2.5–40 μM) and water were used as the standard and blank control, respectively. The results were expressed as TEAC using the Trolox stand curve.

The ORAC assay was performed following a previously described method [18]. Fluorescein was used as the fluorescent indicator of the extent of damage from its reaction with the peroxyl radical derived from AAPH. In brief, in a 96-well plate, 63 nM fluorescein was incubated in phosphate-buffered saline (pH 7.4) in the presence or absence of IDPs or 2-oxo-IDPs (final 2.5–40 μM) at 37 °C for 15 min prior to the addition of AAPH (final 38 mM). Then, fluorescence intensities (excitation at 485 nm and emission at 538 nm) were monitored by an Infinite 200 PRO microplate reader at the end of every cycle (60 s) after shaking for 50 cycles. The oxidant scavenging capacity of 2-oxo-IDPs and IDPs was determined by assessing the area under the fluorescence decay curve (AUC) relative to that of a blank, in which there was water instead of antioxidants. The AUC was calculated using GraphPad Prism software (GraphPad, Inc., La Jolla, CA, USA), and the net AUC was obtained by subtracting the AUC of the blank control (water instead of antioxidants) from that of each sample. Trolox solutions (final 2.5–40 μM) were used for defining the standard curve, and TEAC was calculated by the net AUC of the sample using the Trolox standard curve. 

The iron (Fe^2+^) chelation assay was carried out as described previously [19]. In brief, in a 96-well plate, 5 μL of 2 mM FeCl_2_ and 185 μL of 2-oxo-IDPs or IDPs (final 50–800 μM) were mixed. After 3 min of incubation at room temperature (25 °C), the reaction was inhibited by the addition of 10 μL of 5 mM ferrozine (Dojindo Laboratories), and after another incubation for 10 min, absorbance at 562 nm was measured by an Infinite 200 PRO microplate reader. Ethylenediaminetetraacetic acid (EDTA, final 2.5–40 μM) and water were used as the standard chelator and the blank control, respectively. The Fe^2+^ chelation capacity was defined as described previously [19]: Fe^2+^ chelating capacity (%) = (Ac − As)/Ac × 100, where Ac and As indicate the absorbances of the blank control and sample, respectively. The Fe^2+^ chelation capacity was expressed as EDTA equivalent chelating capacity, which was calculated using the EDTA standard curve.

The reactivity of 2-oxo-IDPs and IDPs to endogenous radicals, such as NO, ONOO^−^, HClO, hydrogen peroxide (H_2_O_2_), and HO^•^, was also confirmed by HPLC with online electrospray ionization-tandem mass spectrometry (HPLC-ESI-MS/MS) analysis. 1-Hydroxy-2-oxo-3-(*N*-methyl-3-aminopropyl)-3-methyl-1-triazene (NOC7, Dojindo Laboratories) was used as a NO donor. ONOO^−^ was synthesized from acidified nitrite and H_2_O_2_ using a quenched-flow method as described previously [20,21]. Contaminating H_2_O_2_ was then decomposed using manganese dioxide. The concentration of ONOO^−^ was determined by means of spectrophotometry at a molar absorption coefficient of ε_302_ =1670 M^−1^ cm^−1^ just before use [20,21]. HO^•^ was generated via the Fenton-like reaction (the CuSO_4_/H_2_O_2_ system) as described previously [22]. In brief, 10 μM 2-oxo-IDPs and IDPs was incubated in 16 mM sodium phosphate buffer (pH 7.4) in the presence or absence of 100 μM radicals at 37 °C for 30 min. After the incubation, the same amount of 0.1% (*v/v*) formic acid was added to the reaction mixture to terminate the reaction, and then the amount of intact 2-oxo-IDPs and IDPs in the mixture was measured by HPLC-ESI-MS/MS analysis as described below. 

### 2.3. pKa Determination by Potentiometric Titration

Potentiometric titration for the determination of the acid dissociation constant (p*K*a) values for 2-oxo-IDPs and IDPs was performed using a F-51 pH meter (HORIBA, Kyoto, Japan) combined with a 9618S-10D Micro ToupH electrode (HORIBA) as described previously [23], with slight modifications. The pH meter was calibrated with standard buffers (HORIBA), i.e., pH 4.01 ± 0.02 (50 mM potassium hydrogen phthalate), pH 6.86 ± 0.02 (25 mM potassium dihydrogenphosphate-dipotassium hydrogenphosphate), and pH 9.18 ± 0.02 (10 mM sodium tetraborate). 2-oxo-IDPs and IDPs were dissolved in ultrapure water and their pH adjusted to 2.0 with 1 M HCl. In brief, the dipeptide solutions (10 mM, 1 mL) were titrated potentiometrically with a dropwise addition of 1 M NaOH at room temperature (25 °C). Sufficient time (about 10–15 s) was allowed to obtain a reasonably stable pH reading before the next base addition. The dipeptide solutions were completely mixed during potentiometric titration with a magnetic stirrer. The p*K*a values were calculated by the second-derivative plot as described previously [23], and are summarized in Supplementary Table S1. 

### 2.4. HPLC Analysis

The purity of commercial carnosine and anserine (2 and 80 mM) was analyzed by HPLC (JASCO, Tokyo, Japan) with a Shodex Asahipak ES-502C 7C column (7.5 × 100 mm; Showa Denko, Tokyo, Japan) under the following conditions: a linear gradient of solvent A (20 mM citrate buffer (pH 5.0)) and solvent B (20 mM citrate buffer (pH 5.0) containing 0.5 M NaCl) (0% B at 0 min; 100% B at 15 min) at a flow rate of 1.0 mL/min. Commercial carnosine and anserine (0.5 and 20 mM) were also analyzed by HPLC with a Scherzo SS-C18 column (2.0 × 50 mm; Imtakt, Kyoto, Japan), as described previously [4], under the following conditions: a linear gradient of solvent A (0.1% formic acid) and solvent B (50% acetonitrile containing 100 mM ammonium formate) (0% B at 0 min; 100% B at 15 min) at a flow rate of 0.3 mL/min. The elution profile was monitored by absorbance at 220 and 250 nm as well as by photodiode array (PDA) detection.

### 2.5. Quantitative HPLC-ESI-MS/MS Analysis

Quantitative HPLC-ESI-MS/MS analysis was performed as described previously [4]. In brief, the samples (final 1 mM) were mixed with stable isotope-labeled 2-oxo-carnosine or 2-oxo-anserine as internal standards (final 500 nM), followed by the quantification of 2-oxo-carnosine and 2-oxo-anserine by HPLC-ESI-MS/MS using the Xevo TQD triple quadrupole mass spectrometer (Waters, MA, USA) coupled with the Alliance e2695 HPLC system (Waters). Samples were separated by the Alliance e2695 system with an Intrada Amino Acid column (2.0 × 50 mm; Imtakt). A discontinuous gradient of solvent A (acetonitrile containing 0.1% formic acid) and solvent B (100 mM ammonium formate) was used as follows: 0% B at 0 min, 60% B at 0.1 min, 70% B at 5 min, 99% B at 9 min, at a flow rate of 0.3 mL/min. Parameters for multiple reaction monitoring were described previously [4].

### 2.6. Purification of 2-Oxo-IDP-Free IDPs

To prepare 2-oxo-carnosine-free carnosine, commercial carnosine standard was fractionated by HPLC using a Scherzo SS-C18 column (10 × 150 mm; Imtakt) and a discontinuous gradient of solvent A (0.1% formic acid) and solvent B (50% acetonitrile containing 100 mM ammonium formate) (0% B at 0 min; 40% B at 0.1 min; 40–70% B at 12 min) at a flow rate of 3 mL/min. Fractions containing carnosine were recovered and referred to as a “purified” sample. 2-oxo-anserine-free anserine was also prepared from commercial anserine standard using the same methodology. Remove of contaminated 2-oxo-IDPs in the purified sample was confirmed by HPLC-ESI-MS/MS analysis.

### 2.7. Statistical Analysis

Data are presented as means ± standard error of the mean (SEM) of at least three independent experiments. Statistical significance was determined by unpaired Student’s *t* test or two-way analysis of variance with Tukey’s multiple comparison test using GraphPad Prism. Values of *p* < 0.05 were considered significant.

## 3. Results

### 3.1. Comparison of Antioxidant Capacity of 2-Oxo-IDPs with IDPs

We investigated the antioxidant capacity of 2-oxo-IDPs (i.e., 2-oxo-carnosine and 2-oxo-anserine) and IDPs (i.e., carnosine and anserine) by multiple in vitro assays: DPPH radical scavenging, FRAP, and ORAC assays (Figure 1B–D). By DPPH radical scavenging assay, a concentration-dependent inhibition of DPPH was observed for 2-oxo-carnosine and 2-oxo-anserine at the concentration range of 10–40 µM, whereas no inhibitory effects by the precursor IDPs (i.e., carnosine and anserine) were observed at the same concentration range (Supplementary Figure S1A). As shown in Figure 1B, 2-oxo-carnosine and 2-oxo-anserine exhibited a greater antioxidant capacity than that of the precursor IDPs (i.e., carnosine and anserine). 

Similarly, the FRAP assay indicated that the absorbance at 596 nm, which indicates the amount of the complex of reduced iron (Fe^2+^)-TPTZ, increased in a 2-oxo-IDPs concentration-dependent manner, while there was no significant increase in the presence of IDPs (Supplementary Figure S1B). As shown in Figure 1C, the TEAC values for 2-oxo-carnosine and 2-oxo-anserine were significantly greater than those for the precursor carnosine and anserine, respectively.

Zhao et al. reported that carnosine exhibited Fe^2+^ chelating ability [24]. Since the FRAP assay relies on the reduction of the complex TPTZ with Fe^3+^ ion to produce the complex TPTZ with Fe^2+^ ion by antioxidants, the Fe^2+^ chelating ability may affect the result obtained from the FRAP assay. We thus performed the Fe^2+^ chelation capacity assay (Supplementary Figure S2). The assay revealed that carnosine and anserine exhibited a significant Fe^2+^ chelation capacity, whereas the Fe^2+^ chelation was not observed in the presence of 2-oxo-IDPs.

We also performed ORAC assay. As shown in Supplementary Figure S1C, the fluorescence decreased in the presence of AAPH, and the AAPH-induced fluorescent decay was dramatically attenuated in the presence of 2-oxo-IDPs but not IDPs. The TEAC values for 2-oxo-carnosine and 2-oxo-anserine were significantly greater than those for the precursor IDPs (Figure 1D). These results demonstrated that the overall antioxidant capacity of 2-oxo-IDPs was greater than that of the corresponding IDPs.

### 3.2. Reactivity of 2-Oxo-IDPs and IDPs to Endogenous Radicals

**Figure 2:**
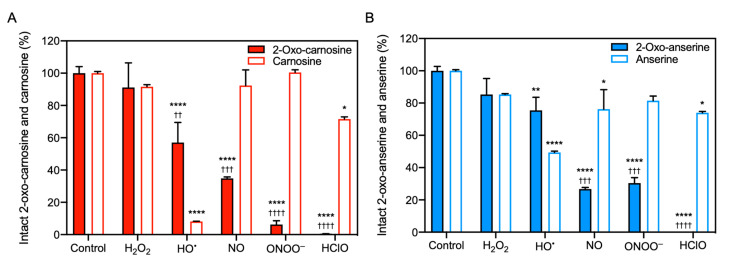
Reactivity of 2-oxo-IDPs and IDPs to radicals. 2-oxo-IDPs and IDPs were incubated in sodium phosphate buffer in the presence or absence of radicals: hydrogen peroxide (H_2_O_2_), and hydroxyl radical (HO^•^) generated by the H_2_O_2_/CuSO_4_ system, nitric oxide (NO) released from 1-hydroxy-2-oxo-3-(*N*-methyl-3-aminopropyl)-3-methyl-1-triazene, peroxynitrite (ONOO^−^), and hypochlorous acid (HClO) at 37 °C for 30 min. The intact dipeptides (**A**), 2-oxo-carnosine and carnosine; (**B**), 2-oxo-anserine and anserine) in the reaction mixture were detected by high performance liquid chromatography with online electrospray ionization-tandem mass spectrometry (HPLC-ESI-MS/MS) analysis. Data are presented as means ± SEM (*n* > 3). * *p* < 0.05, ** *p* < 0.01, **** *p* < 0.0001 versus the respective control; ^††^ *p* < 0.01, ^†††^ *p* < 0.001, ^††††^ *p* < 0.0001 versus the corresponding IDPs under the same condition, compared using two-way analysis of variance with Tukey’s multiple comparison test.

We also evaluated the reactivity of 2-oxo-IDPs and IDPs to endogenous radicals, such as H_2_O_2_, HO^•^, NO, ONOO^−^, and HClO, by HPLC-ESI-MS/MS analysis. The MS analysis revealed that 2-oxo-carnosine and 2-oxo-anserine exhibited a remarkable reactivity to NO, ONOO^−^, and HClO, whereas the corresponding IDPs (carnosine and anserine) showed only a modest reactivity (Figure 2). In contrast, a greater decrease in carnosine and anserine was observed in the presence of HO^•^ generated by the H_2_O_2_/CuSO_4_ system, compared to that in 2-oxo-IDPs. Interestingly, HPLC-ESI-MS/MS analysis revealed that 2-oxo-carnosine and 2-oxo-anserine was generated in the HO^•^-treated carnosine or anserine mixture, respectively (data not shown). A significant decrease in 2-oxo-IDPs and IDPs was not observed in the presence of H_2_O_2_.

### 3.3. Antioxidant Capacity of Commercial IDPs

**Figure 3:**
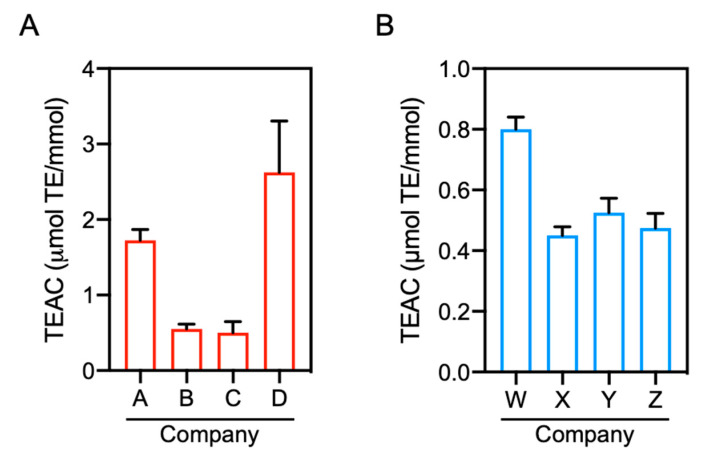
Comparison of the antioxidant capacity of commercial IDPs. The antioxidant capacity was evaluated by DPPH radical scavenging assay in the presence or absence of commercial carnosine (**A**) and anserine (**B**) standards (final 1.25–50 mM). Data are expressed as TEAC and are presented as means ± SEM (*n* = 4).

As described above, positive results of the antioxidant capacity for carnosine and anserine were not observed at the concentration range of 1–40 μM by DPPH radical scavenging and ORAC assays (Figure 1B,D). These results are, however, inconsistent with previous reports. Although previous studies determined the antioxidant capacity of carnosine and anserine using radical scavenging assays with DPPH or 2,2′-azino-bis(3-ethylbenzothiazoline-6-sulfonic acid) and ORAC assay [15,19,25], the assays were performed using higher concentrations (i.e., 0.1–20 mM) of carnosine and anserine and the TEAC values were substantially low. In fact, our preliminary experiment with commercial carnosine standards revealed that a significant antioxidant capacity of carnosine was detected by increasing the dipeptide concentration to 40 mM, and the antioxidant capacity varied by the standard supplier (data not shown). For further investigations of the antioxidant capacity of carnosine and anserine, we performed DPPH radical scavenging assay with commercial standards of carnosine and anserine obtained from four different suppliers. As shown in Figure 3A,B, the antioxidant capacity of carnosine and anserine was detected as previously reported [15]. There were, however, considerable differences in the antioxidant capacity of carnosine and anserine standards among the suppliers. 

### 3.4. Contaminated 2-Oxo-IDPs in Commercial IDPs Standards

**Figure 4:**
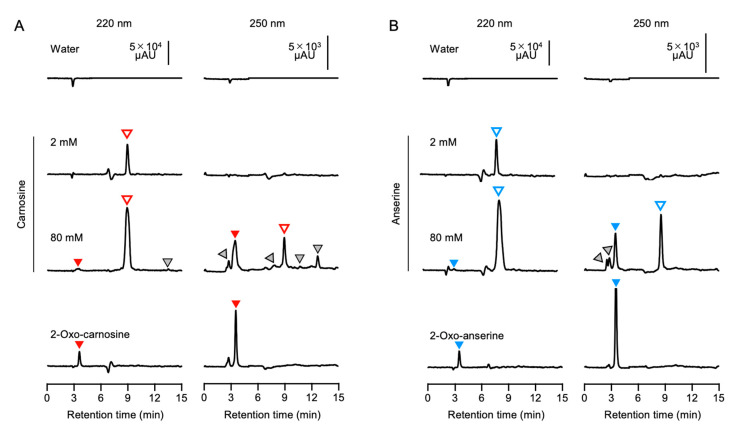
Contaminants including 2-oxo-IDPs in commercial IDPs standards. Different concentrations (2 and 80 mM) of commercial carnosine (**A**) and anserine (**B**) were analyzed by HPLC using an ES-502C 7C column. Representative HPLC chromatograms of the absorbance at 220 nm (left) and 250 nm (right) for the peptide moiety and the 2-oxo-imidazole moiety are indicated. Authentic 2-oxo-carnosine and 2-oxo-anserine (1 mM) were also analyzed by the same approach. Open, filled, and gray triangles indicate IDPs (i.e., carnosine and anserine), 2-oxo-IDPs (i.e., 2-oxo-carnosine and 2-oxo-anserine), and unidentified components, respectively.

We speculated that the difference in the antioxidant capacity for carnosine and anserine standards among the suppliers might be explained by contaminated minor components including 2-oxo-IDPs in the standards. Indeed, a certain amount of contaminants in a commercial carnosine standard was detected by a preliminary HPLC analysis (data not shown). For further investigations of contaminants in the IDPs standards, HPLC analysis was carried out using two different analytical columns (Figure 4). As shown in Supplementary Figure S3, the absorption spectrum of 2-oxo-carnosine and 2-oxo-anserine shifted toward a longer wavelength than that of carnosine and anserine, respectively. We, thus, monitored the absorbance at 220 nm and 250 nm for the peptide moiety and the 2-oxo-imidazole moiety, respectively. By HPLC analysis with a weak cation-exchange ES-502C 7C column, 2 mM carnosine was detected as a single peak at a retention time of 9.1 min in the HPLC chromatogram at 220 nm, with no peaks at 250 nm (Figure 4A). On the other hand, HPLC chromatogram of 80 mM carnosine at 220 nm displayed a major peak of carnosine at a retention time of 9.1 min with a few minor peaks at retention times of 3.6 and 13.6 min, which were also detected by monitoring the absorbance at 250 nm (Figure 4A). Interestingly, the retention time of 3.6 min for a minor peak observed in 80 mM carnosine was consistent with that of synthesized 2-oxo-carnosine. This result indicates that unidentified impurities other than 2-oxo-carnosine also had an absorption at 250 nm. Thus, we performed HPLC analysis with PDA detection (Supplementary Figure S4A). The result revealed that there were unidentified impurities that exhibited absorption spectra at other wavelengths rather than 250 nm in the commercial carnosine standard. Similarly, by HPLC analysis with a multimode (reversed-phase and cation-/anion-exchange) Scherzo SS-C18 column, a few minor peaks were observed in 20 mM carnosine (Supplementary Figure S5A). The retention time of 5.0 min for a minor peak in 20 mM carnosine was consistent with that of synthesized 2-oxo-carnosine (Supplementary Figure S5A). These results strongly suggest that 2-oxo-carnosine may be contaminated in the commercial carnosine standard. 

Likewise, we investigated the contamination of commercial anserine standard with 2-oxo-anserine by the same approach. By HPLC analysis using an ES-502C 7C column, 2 mM anserine standard displayed a single peak at a retention time of 8.7 min at 220 nm, whereas 80 mM anserine displayed several minor peaks as well as a main peak at a retention time of 8.9 min. The retention time (3.5 min) of one of the minor peaks was identical to that of synthesized 2-oxo-ansetine (Figure 4B). As shown in Supplementary Figure S4B, HPLC analysis with PDA detection also revealed that there were impurities that exhibited absorption spectra at other wavelengths rather than 250 nm in the commercial anserine standard. Further, the same results were also obtained when a Scherzo SS-C18 column was utilized for HPLC analysis (Supplementary Figure S5B). These results strongly suggest that 2-oxo-anserine may be contaminated in the commercial anserine standard.

**Figure 5:**
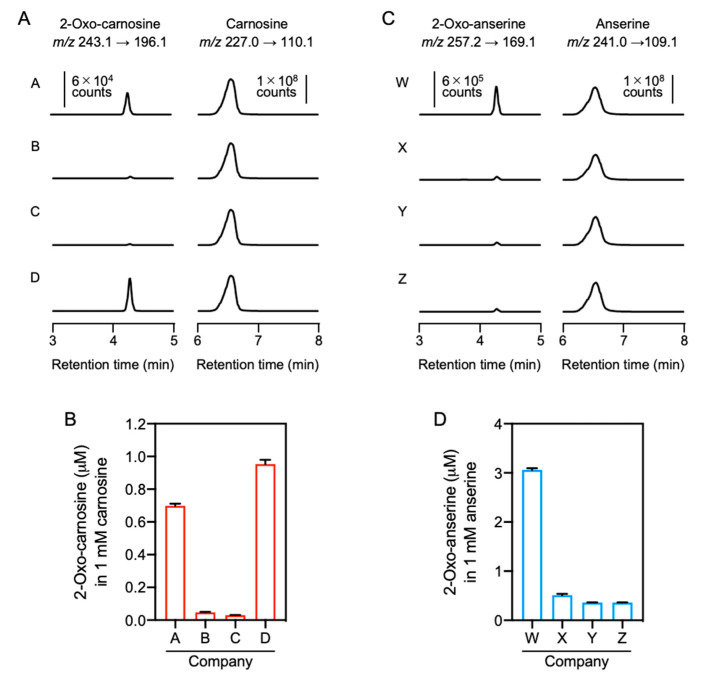
Quantitative HPLC-ESI-MS/MS analysis of 2-oxo-IDPs contamination in commercial IDPs standards. (**A**,**B**) Commercial carnosine (**A**) and anserine (**B**) standards obtained from companies A–D or W–Z, respectively, were analyzed by HPLC-ESI-MS/MS analysis coupled with a stable isotope dilution method. Representative MS/MS chromatograms for 2-oxo-carnosine or 2-oxo-anserine (left) and carnosine or anserine (right) obtained from the four commercial standards are shown. (**C**,**D**) Concentrations of contaminated 2-oxo-carnosine (**C**) or 2-oxo-anserine (**D**) in the commercial carnosine or anserine standard (1 mM). Data are presented as means ± SEM (*n* = 3).

To confirm the contamination of 2-oxo-IDPs in commercial IDPs standards obtained from four different companies, the standards were analyzed by quantitative HPLC-ESI-MS/MS analysis coupled with a stable isotope dilution method [4]. As shown in Figure 5A, 2-oxo-carnosine was detected in all the analyzed carnosine standards, and 2-oxo-carnosine concentrations considerably differed among the standards, ranging from 0.03 to 1.0 µM in 1 mM carnosine (Figure 5B). Interestingly, the 2-oxo-carnosine concentration was strongly correlated with the antioxidant capacity of the carnosine standard (Figure 3A).

HPLC-ESI-MS/MS analysis also revealed that all the analyzed anserine standards were contaminated by 2-oxo-anserine (Figure 5C), and the concentration of 2-oxo-anserine in 1 mM anserine was 0.36–3.1 µM (Figure 5D). The 2-oxo-anserine concentration was correlated with the antioxidant capacity of the anserine standard (Figure 3B). These results strongly suggest that a certain amount of 2-oxo-IDPs contaminated in commercial IDPs standards may contribute to the antioxidant capacity of IDPs standard.

### 3.5. Preparation of Purified IDPs and Analysis of Antioxidant Capacity

**Figure 6:**
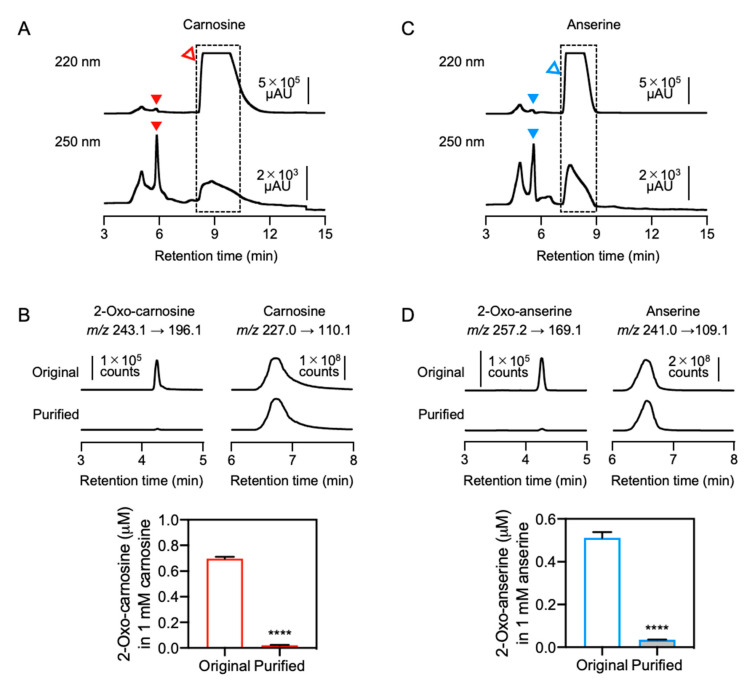
Preparation of purified IDPs. (**A**,**C**) Commercial carnosine and anserine standards were purified by HPLC. The fractions (dashed-line box) were recovered and were referred as “purified” samples. Open and filled triangles indicate IDPs (i.e., carnosine and anserine) and 2-oxo-IDPs (i.e., 2-oxo-carnosine and 2-oxo-anserine), respectively. (**B**,**D**) The concentrations of 2-oxo-carnosine or 2-oxo-anserine in 1 mM original or purified carnosine and anserine samples were analyzed by HPLC-ESI-MS/MS coupled with a stable isotope dilution method. Representative MS/MS chromatograms of 2-oxo-carnosine or 2-oxo-anserine (left), and carnosine or anserine (right) in the original or the purified carnosine and anserine samples. Data are presented as means ± SEM (*n* = 3). **** *p* < 0.0001 versus the original sample, compared using unpaired Student’s *t* test.

To further examine effects of contaminants in commercial IDPs on the antioxidant capacity, contaminants including 2-oxo-IDPs were removed from commercial IDPs standards by HPLC (Figure 6). As shown in Figure 6A, carnosine was completely separated from 2-oxo-carnosine under the HPLC condition. Carnosine eluted at a retention time of 8–11 min was recovered and was referred as “purified” sample. The minor peaks observed in the original sample were not detected in the purified sample (Supplementary Figure S6A,C). A significant decrease of contaminated 2-oxo-carnosine was confirmed by quantitative HPLC-ESI-MS/MS analysis (Figure 6B). However, unpredictably, a detectable amount of 2-oxo-carnosine (0.0022%) was still observed in the purified carnosine. We examined whether 2-oxo-carnosine could be formed by an autooxidation of carnosine. As shown in Supplementary Figure S7, 2-oxo-carnosine was formed by the autooxidation of carnosine during the purification process. Thereby, the minor but detectable amount of 2-oxo-carnosine in the purified sample was presumed to be due to the autooxidation of carnosine to form 2-oxo-carnosine during the purification process. 

Anserine was also purified from a commercial anserine standard by the same approach. After separation by HPLC, the peak corresponding to anserine at a retention time of 7.2–9.2 min was recovered and was referred as the “purified” sample (Figure 6C). The minor peaks in the original sample were not observed in the purified sample (Supplementary Figure S6B,D). A significant decrease in 2-oxo-anserine contamination was confirmed by quantitative HPLC-ESI-MS/MS analysis (Figure 6D). However, a detectable amount of 2-oxo-anserine (0.0036%) was still observed in the purified anserine, which is also presumed to be due to the autooxidation of anserine during the purification process.

**Figure 7:**
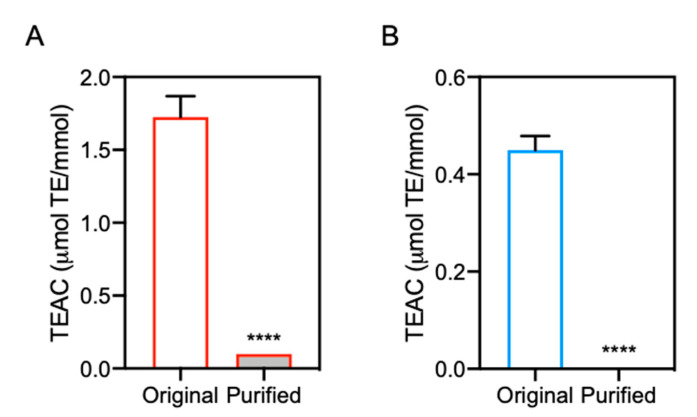
Antioxidant capacity of purified IDPs. The antioxidant capacity of the purified carnosine (**A**) and anserine (**B**) was evaluated by DPPH radical scavenging assay and was compared with that of the original samples, respectively. Data are expressed as TEAC and are represented as means ± SEM (*n* = 4). **** *p* < 0.0001 versus the original sample, compared using unpaired Student’s *t* test.

The antioxidant capacity of the original and purified IDPs was evaluated by DPPH radical scavenging assay. As shown in Figure 7, the antioxidant capacity of purified carnosine and anserine was significantly lower than that of the original carnosine and anserine, respectively. These results suggested that contaminated 2-oxo-IDPs may predominantly contribute to the antioxidant capacity of commercial IDPs standards. 

## 4. Discussion

Since the antioxidant capacity of IDPs was discovered in the 1980s, numerous studies of the physiological effects of IDPs have been performed (reviewed in [2]). Recently, we provided the first evidence for the endogenous generation of 2-oxo-carnosine, 2-oxo-anserine, 2-oxo-homocarnosine, and 2-oxo-homoanserine in vivo [4,16]. Further, DPPH radical scavenging assay revealed that 2-oxo-carnosine exhibited a remarkably greater antioxidant capacity than that of the precursor carnosine [4]. For the evaluation of the antioxidant capacity of 2-oxo-IDPs, in the current study, we employed three different in vitro antioxidant assays: DPPH radical scavenging, FRAP, and ORAC assays. These three assays are classified as three groups: an electron transfer (ET)-based assay (i.e., FRAP assay), a hydrogen atom transfer (HAT)-based assay (i.e., ORAC assay), and a mixed-mode (ET/HAT-based) assay (i.e., DPPH radical scavenging assay), respectively [26,27]. ET-based assays measure the capacity of an antioxidant in the reduction of an oxidant, which changed color when the oxidant was reduced. HAT-based assays measure the capacity of an antioxidant to quench peroxyl radicals, generated by AAPH in this study, by hydrogen atom donation. Although these three assays are based on different mechanisms, all the in vitro assays employed in the current study demonstrated that the antioxidant capacity of 2-oxo-IDPs (i.e., 2-oxo-carnosine and 2-oxo-anserine) was significantly greater than that of the precursor IDPs (i.e., carnosine and anserine). These results suggest that the oxidized modification at the C-2 position of the imidazole group of 2-oxo-IDPs may contribute to the potent antioxidant capacity.

We also examined the Fe^2+^ chelating capacity, which is considered as an important property for the antioxidant capacity because Fe^2+^ catalyzes the generation of HO^•^ via the Fenton reaction [28]. The assay revealed that carnosine and anserine efficiently chelated Fe^2+^, which is consistent with recent studies that carnosine exhibited Fe^2+^ chelation capacity [24,25]. However, Ishihara et al. reported that the Fe^2+^ chelation capacity of anserine was significantly lower than that of carnosine [25], which is inconsistent with the current result. For careful investigation of the difference in the Fe^2+^ chelation capacity of carnosine and anserine, further studies with highly purified IDPs standards are required. In contrast, we discovered for the first time that the Fe^2+^ chelation capacity of 2-oxo-IDPs was nearly abolished. Previous studies suggest that the metal chelating capacity of carnosine is dependent on pH [29,30]. Torreggiani et al. reported that the imidazole ring of carnosine in its neutral form existed at neutral pH in equilibrium between the two tautomeric forms: the Nπ protonated form and the Nτ protonated form [31]. Further, they reported that the Nπ and Nτ nitrogen atoms of the imidazole ring of carnosine were involved in the formation of Cu^2+^- and Zn^2+^-carnosine complexes [31,32]. The potentiometric titration revealed that 2-oxo-IDPs and IDPs showed similar p*K*a values for the carboxylic group of the histidine residue and the amino group of the β-alanine residue, respectively (Supplementary Table S1). Interestingly, the p*K*a values for the imidazole ring of carnosine and anserine were similar, whereas those for 2-oxo-IDPs were not determined. These results suggest that the mono-oxygenation at the C-2 position of the imidazole group of IDPs may affect the ionization state at neutral pH, resulting in the disappearance of the Fe^2+^ chelation capacity of 2-oxo-IDPs. Further studies to clarify the impact of the oxidization modification of 2-oxo-IDPs on the Fe^2+^ chelation capacity are required.

Conflicting results have been reported regarding the antioxidant capacity levels of IDPs. For example, it has been reported that carnosine exhibits a greater antioxidant capacity than that of anserine [33], while other studies have reported that anserine exhibits a higher antioxidant capacity than that of carnosine [34,35]. Some studies have shown that carnosine and anserine inhibit peroxidation (reviewed in [36]), whereas another study has reported that carnosine and anserine exhibits only a modest inhibitory effects on peroxidation [37]. We consider two possibilities to explain this inconsistency, at least in part. One is that there are antioxidant impurities in IDPs standards. Indeed, in the current study, some unidentified peaks in addition to a peak of 2-oxo-IDPs were observed in 80 mM carnosine and anserine standards by monitoring the absorbance at 250 nm. Further, HPLC analysis with PDA detection revealed that there were unidentified impurities that exhibited absorption spectra at other wavelengths rather than 250 nm in the commercial carnosine and anserine standards (Supplementary Figure S4). A previous study demonstrated that small amounts of hydrazine (0.01–0.20%), a strong reductant, were present in various sources of commercial carnosine, thereby interfering with lipid oxidation [38]. These results suggest that impurities in the standards could affect the antioxidant capacity of IDPs and the protective effects of IDPs against various oxidative stress-related diseases previously reported [39,40,41,42,43,44,45].

The other is the endogenous conversion of IDPs to 2-oxo-IDPs. We previously reported that 2-oxo-IDPs was generated in vivo depending on oxidative stress [4]. Further, using HPLC-ESI-MS/MS analysis, we herein revealed for the first time that the reactivity of 2-oxo-IDPs to endogenous radicals, such as NO, ONOO^−^, and HClO, was significantly greater than that of the precursor carnosine and anserine. In contrast, carnosine and anserine exhibited a greater reactivity to HO^•^, generated by the H_2_O_2_/CuSO_4_ system, than did 2-oxo-carnosine and 2-oxo-anserine. A previous study via electron paramagnetic resonance also demonstrated that carnosine efficiently scavenged HO^•^ [46]. Interestingly, HPLC-ESI-MS/MS analysis revealed that 2-oxo-carnosine and 2-oxo-anserine was generated in the reaction mixture of carnosine and anserine with HO^•^, respectively (data not shown). Further, our preliminary experiments showed that 2-oxo-carnosine formation by the reaction of carnosine and HO^•^ was not dependent on the incubation time and the concentration of H_2_O_2_ and CuSO_4_ (data not shown), presumably because the formed 2-oxo-carnosine could react with excess HO^•^ generated by the H_2_O_2_/CuSO_4_ system. These results suggest that IDPs can first be converted to 2-oxo-IDPs by the reaction with endogenous radicals such as HO^•^, and the formed 2-oxo-IDPs can exhibit strong scavenging activities against other endogenous radicals, such as NO, ONOO^−^, and HClO. In fact, our previous study demonstrated that the overexpression of carnosine synthase increased 2-oxo-carnosine production in H_2_O_2_- or rotenone-treated SH-SY5Y cells, resulting in the cytoprotection from the oxidative stress-induced cell death [4]. Taken together, the conversion of IDPs to 2-oxo-IDPs may be critical for the protective effects of IDPs against the various oxidative stress-related diseases previously reported [39,40,41,42,43,44,45].

In this study, quantitative HPLC-ESI-MS/MS analysis revealed that all the analyzed commercial carnosine and anserine standards were contaminated with 2-oxo-carnosine (0.003–0.01%) and 2-oxo-anserine (0.04–0.3%), respectively. Interestingly, there was a strong correlation between 2-oxo-IDP contamination levels and the antioxidant capacity. Our finding that 2-oxo-IDPs exhibits a greater antioxidant capacity than does the precursor IDPs suggests that the contamination of 2-oxo-IDPs in the commercial IDPs standards may in part explain the apparent antioxidant capacity of commercial IDPs. In support of this hypothesis, we found that the elimination of contaminated 2-oxo-IDPs from the IDPs standards caused the decrease in the antioxidant capacity. Given these findings indicating that 2-oxo-IDPs contamination affects the antioxidant capacity of commercial IDPs, further studies with highly purified IDPs are needed to re-evaluate biological and chemical properties of IDPs.

As mentioned above, we successfully prepared IDPs with high purity by HPLC purification; however, HPLC-ESI-MS/MS revealed that 2-oxo-IDPs was not eliminated and a minor but detectable amount of 2-oxo-IDPs remained in the purified samples (2-oxo-carnosine: 0.0022% and 2-oxo-anserine: 0.0036%, respectively). The slow formation of 2-oxo-IDPs can occur via an autooxidation of IDPs catalyzed by metals such as Cu^2+^ during purification (Supplementary Figure S6). Further preparative processes or storage may result in some degree of contamination with 2-oxo-IDPs in IDP standards. 

In our previous study, an in vitro analysis using a histidine analog *N*-benzoyl-histidine demonstrated that *N*-benzoyl-histidine was initially oxidized at the C-2 position of the imidazole ring to form *N*-benzoyl-2-oxo-histidine by a free radical-generating system (copper/ascorbate), and the formed *N*-benzoyl-2-oxo-histidine was subsequently oxidized to form a series of further oxidized products via the ring opening reaction of the imidazole ring [47]. The mono-oxigenized modification of histidine was also detected on various polypeptides, including copper/ascorbate-treated bovine serum albumin or β-amyloid peptide, and ultraviolet C-irradiated immunoglobulin gamma 1 [48,49,50,51], and a similar degradation pathway of the 2-oxo-histidine residue has been proposed [51,52]. Thus, it is possible that the commercial carnosine and anserine may contain contaminants other than 2-oxo-forms, which are not detected in the experimental conditions used in this study. Indeed, our preliminary experiments also showed that 2-oxo-carnosine was degraded to a series of further oxidized products via the ring opening reaction of the imidazole ring (data not shown). These breakdown products of 2-oxo-IDPs may affect the antioxidant capacity. However, the detailed mechanism underlying 2-oxo-IDPs decomposition and the contribution of degraded products of 2-oxo-IDPs via reactions with radicals to the antioxidant capacity remains unclear and further studies are required. 

Various studies have demonstrated the beneficial effects of dietary supplementation with carnosine or anserine on various animal models of human diseases, including a streptozotocin-induced diabetic retinopathy rat model [53]; a cirrhotic rat model by bile duct ligation [54]; an ethanol-induced chronic liver injury in mice [55]; a focal ischemia rat model [39]; and a transgenic mice model of Alzheimer’s disease [40]. Further, to date, multiple double-blind, placebo-controlled, randomized controlled clinical trials have been conducted with oral carnosine supplementation. For example, dietary supplementation with carnosine has been used to ameliorate syndromes in patients with Parkinson disease [41] and gastric ulcers [56]. In addition, oral administration of carnosine or anserine for 12 weeks resulted in a significant improvement of oxidative stress, glycemic control, and renal function in pediatric patients with diabetic nephropathy [42]; a significant decrease in risk factors of type-2 diabetes, such as fat mass, fasting blood glucose, and serum triglycerides [43]; and protection of elderly patients with mild cognitive impairment from cognitive decline [44]. Furthermore, the beneficial effects of supplementation with anserine plus carnosine on metabolic, neurological, immunological, cardiovascular, and renal functions have been demonstrated by clinical trials using a chicken meat extract, which contains anserine and carnosine at a ratio of 3:1 or 2:1 [45,57,58,59,60]. We previously demonstrated that 2-oxo-carnosine, but not the precursor carnosine, significantly protected human neuroblastoma SH-SY5Y cells from rotenone-induced oxidative stress [4]. However, the biological relevance of 2-oxo-IDPs, especially in vivo, remains unclear, and further studies with animal experiments as well as clinical trials, are needed.

## 5. Conclusions

In the present study, we evaluated the antioxidant capacity of 2-oxo-carnosine and 2-oxo-anserine. Furthermore, we identified 2-oxo-carnosine and 2-oxo-anserine contamination in commercial carnosine and anserine standards, respectively, which affected the antioxidant capacity. By using 2-oxo-IDPs and purified IDPs, we obtained two key results. (1) 2-oxo-carnosine and 2-oxo-anserine exhibited a greater antioxidant capacity than that of carnosine and anserine, respectively. (2) Highly purified carnosine and anserine exhibited only a modest antioxidant capacity. These findings suggest that 2-oxo-IDPs is a main driver of the overall antioxidant capacity of IDPs. Since 2-oxo-IDPs is endogenously produced in vivo under normal conditions and their levels are elevated under oxidative stress, the conversion of IDPs to 2-oxo-IDPs may be critical for protection against oxidative stress-associated diseases in vivo. These results provide new insight in the biological relevance and antioxidant effects of IDPs. Further works are needed to determine the detailed mechanism underlying the antioxidant capacity of 2-oxo-IDPs. 

## Data Availability

Data are contained within the article and supplementary material.

## Supplementary Materials

The following are available online at https://www.mdpi.com/article/10.3390/antiox10091434/s1, Figure S1: Evaluation of the antioxidant capacity of 2-oxo-IDPs and IDPs by multiple in vitro antioxidant assays, Figure S2: Iron (Fe^2+^) chelation capacity of 2-oxo-IDPs and IDPs, Figure S3: Ultraviolet absorption spectrum of 2-oxo-IDPs and IDPs, Figure S4: PDA detection of commercial IDPs standards, Figure S5: Contaminants in commercial carnosine and anserine standard, Figure S6: HPLC analysis of purified carnosine and purified anserine, Figure S7: Formation of 2-oxo-carnosine via autooxidation, Table S1: p*K*a values of 2-oxo-IDPs and IDPs.
