# Supplementary material for: 2-Oxo-Imidazole-Containing Dipeptides Play a Key Role in the Antioxidant Capacity of Imidazole-Containing Dipeptides
Source: Antioxidants (Basel). 2021 Sep 8;10(9):1434. doi: 10.3390/antiox10091434 (PMC8466808; doi:10.3390/antiox10091434)
Supplement: Supplementary file 1 [file antioxidants-10-01434-s001.zip › antioxidants-1372883- proof done supp.pdf]

2-Oxo-Imidazole-Containing Dipeptides Play a Key Role in the Antioxidant Capacity of Imidazole-Containing Dipeptides

Shingo Kasamatsu [1,†], Somei Komae [1,†], Kana Matsukura [1,†], Yuki Kakihana [1], Koji Uchida [2], and Hideshi Ihara [1,*]

[1] Department of Biological Science, Graduate School of Science, Osaka Prefecture University, Osaka 599-8531, Japan

[2] Graduate School of Agricultural and Life Sciences, The University of Tokyo, Tokyo 113-8657, Japan.

[†] These authors contributed equally to this work.

[*] Correspondence: Hideshi Ihara, Department of Biological Science, Graduate School of Science, Osaka Prefecture University, 1-1 Gakuen-cho, Sakai, Osaka 599-8531, Japan. Tel.: +81-72-254-9753; E-mail: ihara@b.s.osakafu-u.ac.jp.

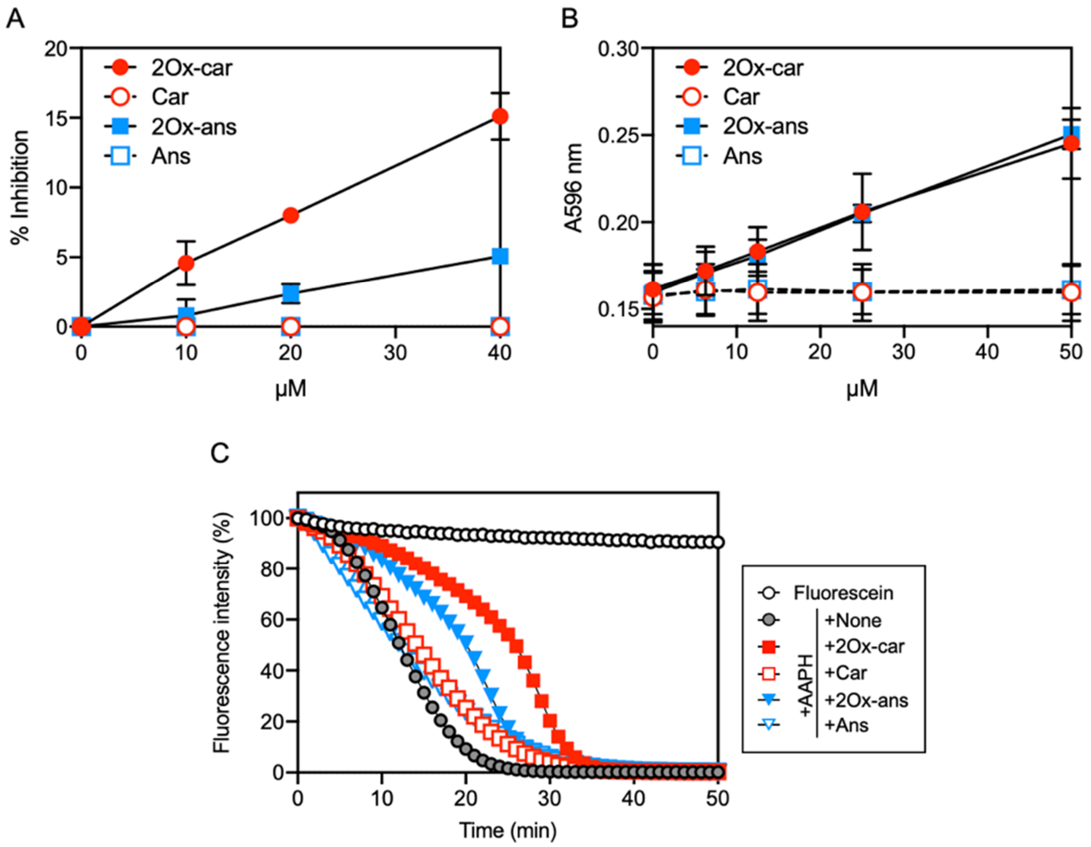

Supplementary Figure S1. Evaluation of the antioxidant capacity of 2-oxo-IDPs and IDPs by multiple in vitro antioxidant assays. (**A, B**) Percent inhibition of 2,2-diphenyl-1-picrylhydrazyl radical (**A**) and ferric reducing/antioxidant power (**B**) in the presence of 2-oxo-carnosine (2Ox-car, filled red circle), carnosine (Car, open red circle), 2-oxo-anserine (2Ox-ans, filled blue square), and anserine (Ans, open blue square). Data are presented as means ± standard error of the mean (SEM) (n > 4). (**C**) Representative fluorescence decay curves during oxygen radical absorbance capacity assay in the presence of 2-oxo-carnosine (2Ox-car, filled red square), carnosine (Car, open red square), 2-oxo-anserine (2Ox-ans, filled blue triangle), and anserine (Ans, open blue triangle). Fluorescein (open circle): the positive control which was incubated without 2,2'-azobis(2-methylpropionamidine) dihydrochloride (AAPH) and dipeptides; AAPH (filled gray circle): the blank control treated with AAPH in the absence of dipeptides.

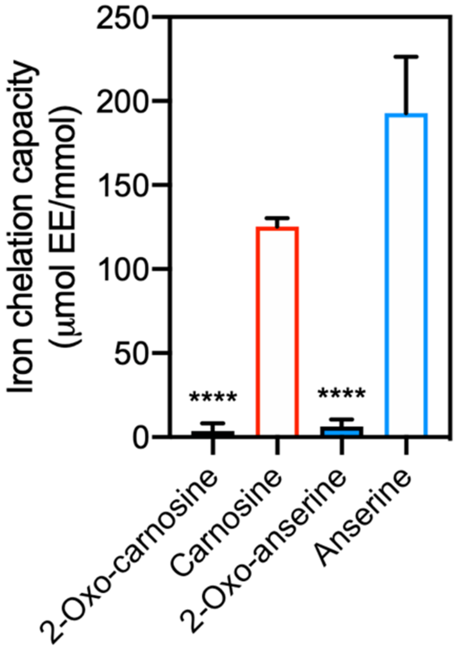

Supplementary Figure S2. Iron (Fe^{2+}) chelation capacity of 2-oxo-IDPs and IDPs. Iron (Fe^{2+}) chelation capacity of 2-oxo-IDPs and IDPs was evaluated using ferrozine. The Fe^{2+} chelation capacity was expressed as ethylenediaminetetraacetic acid (EDTA) equivalent chelating capacity: μmol EDTA equivalent (EE) per mmol samples. Data are presented as means ±SEM ($n > 4$). ****$p < 0.0001$ versus the corresponding IDPs, compared using unpaired Student's t test.

.

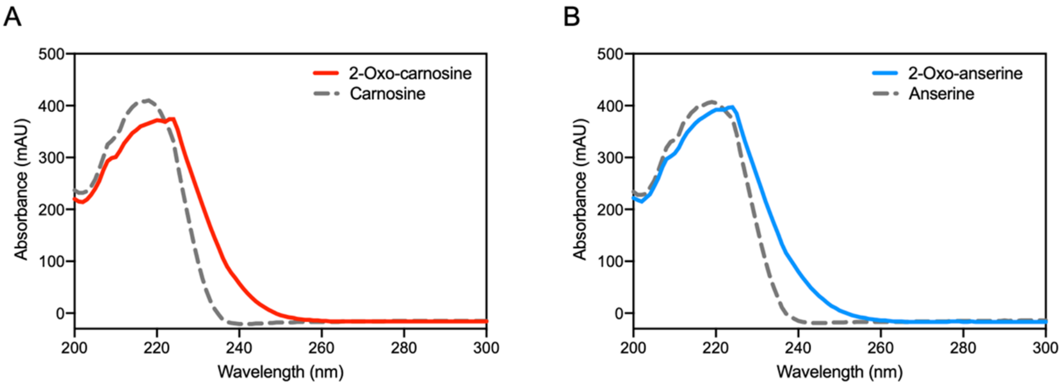

Supplementary Figure S3. Ultraviolet absorption spectrum of 2-oxo-IDPs and IDPs. The ultraviolet absorption spectrum of 2-oxo-imidazole-containing dipeptides (2-oxo-IDPs; 2-oxo-carnosine (**A**) and 2-oxo-anserine (**B**)) was shifted toward a longer wavelength than that of the precursor imidazole-containing dipeptides (IDPs; carnosine and anserine), respectively.

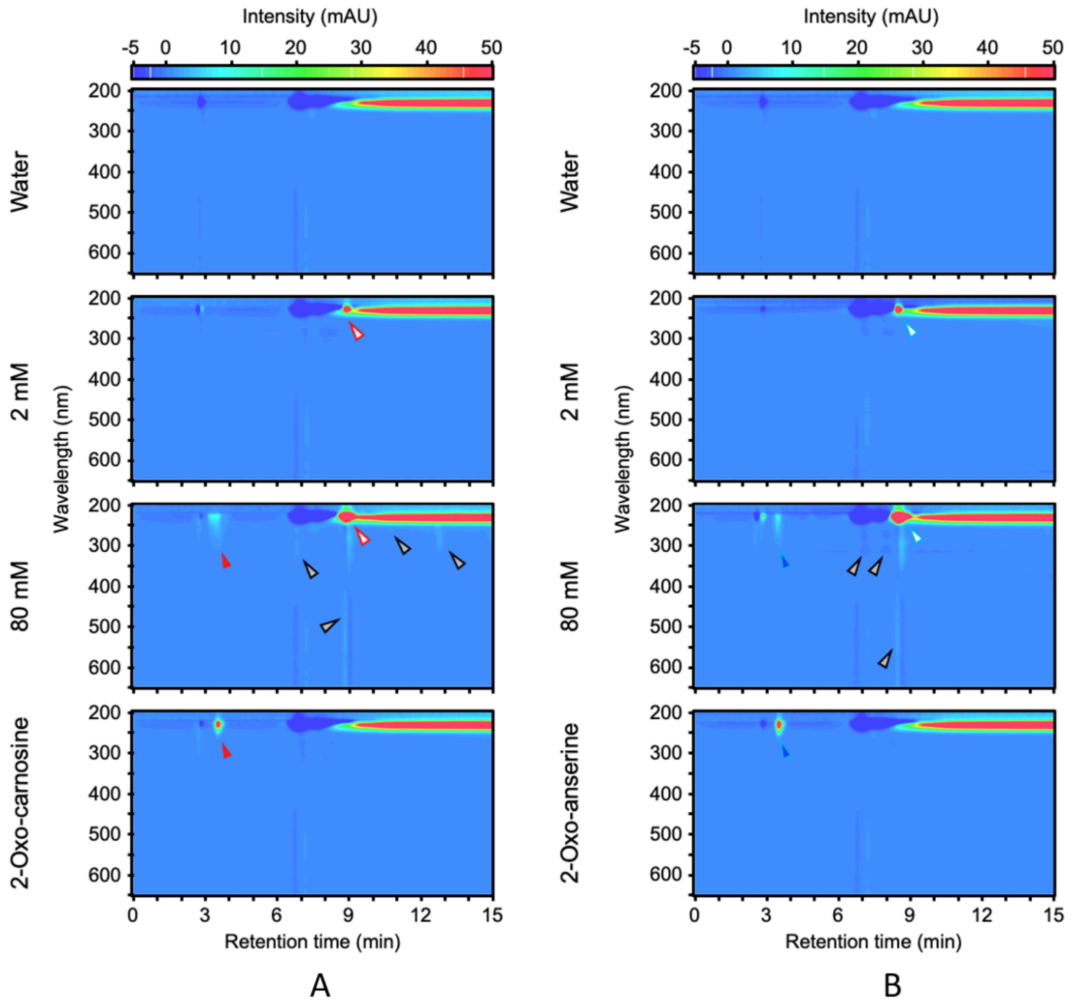

Supplementary Figure S4. PDA detection of commercial IDPs standards. Contaminants including 2-oxo-IDPs in commercial IDPs standards. Different concentrations (2 and 80 mM) of commercial carnosine (**A**) and anserine (**B**) were separated by high performance liquid chromatography (HPLC) using an ES-502C 7C column and the absorption spectra were recorded by a photodiode array (PDA) detector. Authentic 2-oxo-carnosine and 2-oxo-anserine (1 mM) were also analyzed by the same approach. Open, filled, and gray triangles indicate IDPs (i.e., carnosine and anserine), 2-oxo-IDPs (i.e., 2-oxo-carnosine and 2-oxo-anserine), and unidentified components, respectively.

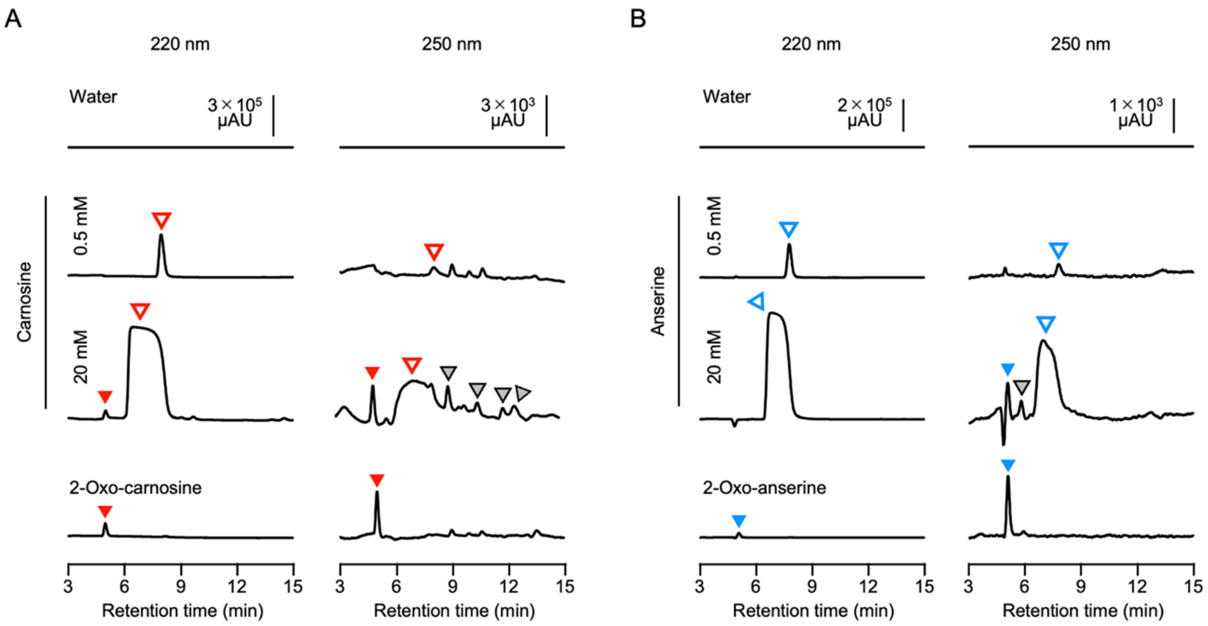

Supplementary Figure S5. Contaminants in commercial carnosine and anserine standard. Different concentrations (0.5 and 20 mM) of commercial carnosine (**A**) and anserine (**B**) standards were analyzed by HPLC with a multi-mode (reversed-phase and cation-/anion-exchange) Scherzo SS-C18 column. Representative HPLC chromatograms of the absorbance at 220 nm (left) and 250 nm (right) were indicated. Authentic 2-oxo-carnosine and 2-oxo-anserine were analyzed by the same approach. Open, filled, and gray triangles indicate IDPs (i.e., carnosine and anserine), 2-oxo-IDPs (i.e., 2-oxo-carnosine and 2-oxo-anserine), and unidentified components, respectively.

.

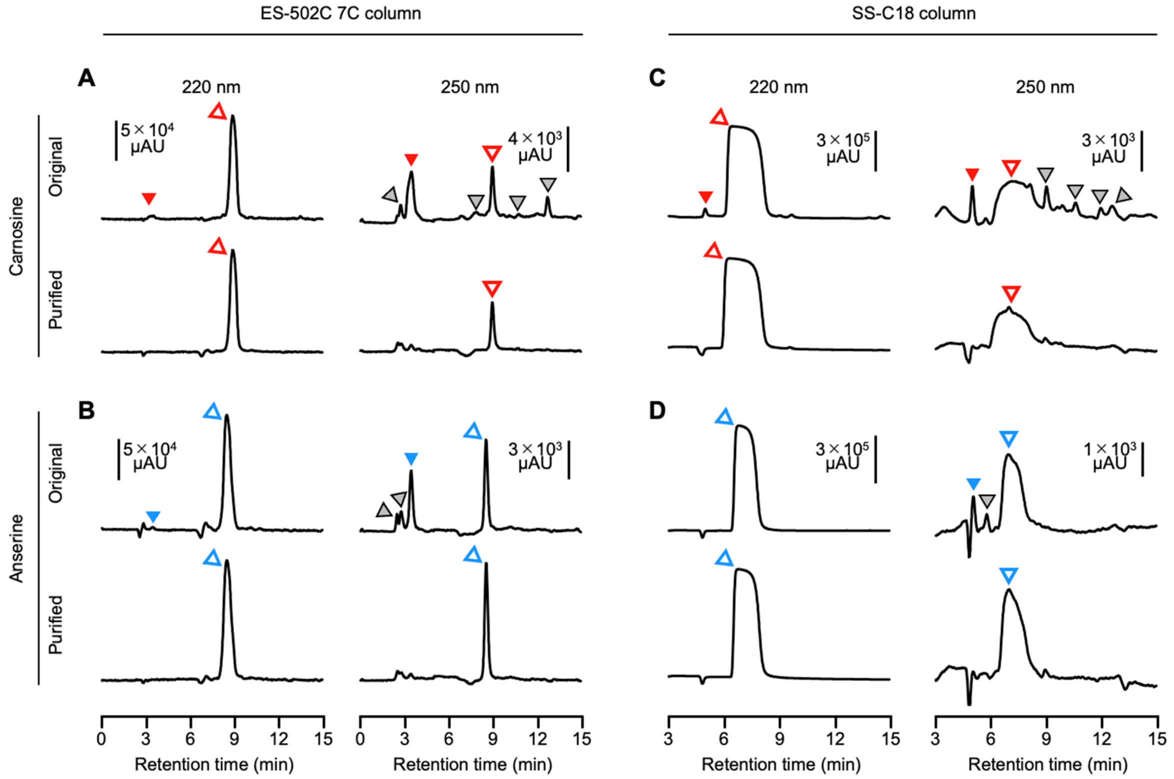

Supplementary Figure S6. HPLC analysis of purified carnosine and purified anserine. Original and purified carnosine and anserine samples were analyzed by HPLC using a weak cation-exchange ES-502C 7C column (**A, B**) or a multi-mode Scherzo SS-C18 column (**C, D**). Representative HPLC chromatograms of the absorbance at 220 nm (left) and 250 nm (right) were indicated. Open, filled, and gray triangles indicate IDPs (i.e., carnosine and anserine), 2-oxo-IDPs (i.e., 2-oxo-carnosine and 2-oxo-anserine), and unidentified components, respectively.

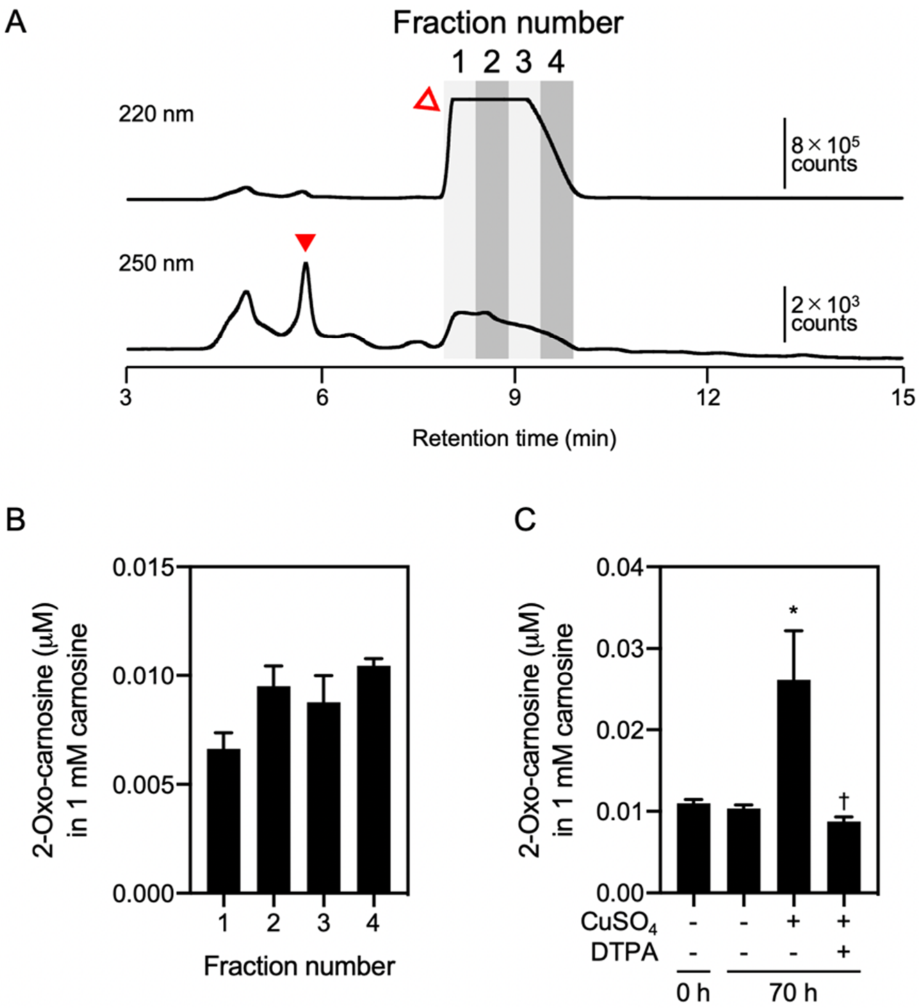

Supplementary Figure S7. Formation of 2-oxo-carnosine via autooxidation. (**A**) Commercial carnosine was separated by HPLC using an ES-502C 7C column. Representative HPLC chromatograms of the absorbance at 220 nm and 250 nm were indicated. Fractions 1–4 were recovered, referred to "purified" sample, and utilized for further experiments mentioned below. Filled and open arrowheads indicate carnosine and 2-oxo-carnosine, respectively. (**B**) 2-oxo-carnosine in each fraction was quantified by HPLC with online electrospray ionization-tandem mass spectrometry (HPLC-ESI-MS/MS) analysis with a stable isotope dilution method. Data are presented as means ± SEM (n = 3). (**C**) Formation of 2-oxo-carnosine via a metal-dependent autooxidation. Purified carnosine (2 mM) was incubated in the presence or absence of 2 mM $CuSO_4$ with or without 4 mM diethylenetriaminepentaacetic acid (DTPA) in 20 mM sodium phosphate buffer (pH 7.0) for 70 h at 4°C. The concentration of 2-oxo-carnosine was determined by quantitative HPLC-ESI-MS/MS analysis coupled with a stable isotope dilution method. Data are presented as means ± SEM (n = 3). *$p < 0.05$ versus the control; †$p < 0.05$ versus the $CuSO_4$-treated group, compared using one-way analysis of variance with Tukey's multiple comparison test.

Supplementary Table S1. p*K*a values of 2-oxo-IDPs and IDPs.

	**p*K*a values for**		
	Carboxyl group	Imidazole ring	Amino group
2-oxo-carnosine	2.47	N.D.	9.48
Carnosine	2.45	6.82	9.52
2-oxo-anserine	2.50	N.D.	9.67
Anserine	2.42	7.07	9.66

N.D., not determined.
